# Mesenchymal Stromal Cell-Based Therapy

**DOI:** 10.3390/cells12040559

**Published:** 2023-02-09

**Authors:** Dina Mönch, Marlies E. J. Reinders, Martin J. Hoogduijn, Marc-Hendrik Dahlke

**Affiliations:** 1 Dr. Margarete Fischer-Bosch Institute of Clinical Pharmacology, 70376 Stuttgart, Germany; 2 University of Tübingen, 72074 Tübingen, Germany; 3 Erasmus MC Transplant Institute, Department of Internal Medicine, Erasmus University Medical Center, 3015 GD Rotterdam, The Netherlands; 4 Department of Surgery, Robert-Bosch-Hospital, 70376 Stuttgart, Germany

The use of mesenchymal stromal cells (MSCs) for clinical application is intensively investigated for a variety of areas, such as bone repair, haematological and autoimmune diseases, and solid organ transplantation. Moreover, increasing insights into their mechanism of actions revealed a lively (paracrine) interaction between MSCs and recipient (immune) cells and identified novel mediators such as extracellular vesicles (EVs). Both paracrine signals and EVs have been shown to play a role in the effects of MSCs and may potentially complement MSC-based therapies or represent therapeutic alternatives. This Special Issue of *Cells* is devoted to the many aspects of MSC-based therapy and includes original data papers and reviews on preclinical models, clinical studies, as well as MSC manufacturing protocols.

There are still limited options for functional rehabilitation after ischemic stroke. Early approaches using MSCs have been shown to be unable to replace dead neurons after stroke events in a preclinical setting, but other features of MSCs, such as the secretion of growth factors at the injured site, the promotion of axonal outgrowth, neuro- and angiogenesis, and synaptic remodeling, have been identified in recent years as key mediators of their therapeutic effect. Another treatment option commonly used for stroke recovery is physical exercise (PE). PE ameliorates motor dysfunction, improves neuroplasticity, and decreases cognitive decline in the brain. Nucci et al. investigated the effect of MSC-based cell therapy and physical exercise training in a murine stroke model using noninvasive molecular imaging techniques. The combination of local MSC treatment and PE resulted in the improved execution of complex movements and a faster recovery of symmetry over time [1]. Moreover, MSC tracking showed an increased signal at the damaged site, indicating an increase in cell number at the site, especially during the acute phase of stroke [1,2].

Systemically delivered MSCs for ischemic stroke therapy would have to cross the blood–brain barrier (BBB) in order to reach their site of action and induce their therapeutic effects. Yarygin et al. reviewed the available data on intra–arterial MSC administration for the intra-cerebral delivery of MSCs. They concluded that administered MSCs either temporarily attach to the walls of the cerebral vessels and ultimately return to the blood stream or penetrate the BBB, resulting in homing in the perivascular space and deeper migration into the parenchyma [3].

The preconditioning of MSCs prior to in vivo administration is a promising opportunity to enhance their therapeutic efficacy. The preconditioning of MSCs by modifying their physical environment, chemical or pharmaceutical agents, bioactive factors, or specific gene manipulation could prolong survival and improve the function of immunomodulatory effects. The review of Cheng provides insights into different preconditioning strategies aimed at optimizing MSCs for inhibiting allograft rejection. It discusses novel directions, such as the simultaneous transfection of multiple genes or the ex vivo treatment of the allograft before implantation for further improvements [4].

This Special Issue also covers the important topics of MSC manufacturing guidelines and storage. According to European regulation 1394/2007, MSCs are an advanced therapy medicinal product that must be produced following good manufacturing practice (GMP) standards. This requires strictly organized protocols for staff organization; premises/equipment qualification and monitoring; raw material management; starting materials; technical manufacturing processes; quality controls; and the release, thawing and infusion of MSCs [5]. Lechanteur et al. comprehensively describe how they adapted their existing clinical-grade MSC production process to a full GMP-compliant set-up, which included validating the ex vivo culture, as well as demonstrating the short-term and long-term stability of fresh and thawed MSCs. They also validated a variant of the process, specifically for the preparation of fresh MSCs for local injection in the treatment of Crohn’s disease [5].

Not only are manufacturing protocols important factors that can affect MSC viability and subsequent efficacy before their actual application, but cell storage media and MSC preservation are too. Ścieżyńska et al. reviewed the influence of hypothermic storage fluids on MSC stability. They conclude that MSCs need to recover after cryopreservation, prior to administration, to optimize in vivo survival as well as immunomodulatory and therapeutic properties [6]. However, only 25% of the clinical studies involving MSCs provide data on manufacturing parameters or product viability, which makes it difficult to set up universal guidelines, underscoring the need for the rigorous testing of optimal manufacturing conditions. 

The review presented by Ścieżyńska et al. summarizes a problem of the MSC field in general. Today, there are more than 75,000 publications on MSCs and this ever-expanding flood of articles makes it hard to extract findings that move the field further towards the development of MSC therapy with clinical efficacy. 

It will be helpful to encourage the publication of negative studies in the field to form a balanced view of the therapeutic possibilities and impossibilities of MSCs. In addition, it will be necessary to re-think the evaluation of published work solely via impact factors by adding a reproducibility factor as a quality indicator [7]. In such a setting, manuscripts that convincingly confirm another study could obtain priority for publication, adding to a more solid foundation for future clinical trials.
